# The combined influence of rootstock and vintage climate on the grape and wine flavonoids of *Vitis vinifera* L. cv. Cabernet Sauvignon in eastern China

**DOI:** 10.3389/fpls.2022.978497

**Published:** 2022-08-16

**Authors:** Xiao Han, Yu Wang, Hao-Cheng Lu, Hang-Yu Yang, Hui-Qing Li, Xiao-Tong Gao, Xuan-Xuan Pei, Fei He, Chang-Qing Duan, Jun Wang

**Affiliations:** ^1^Center for Viticulture and Enology, College of Food Science and Nutritional Engineering, China Agricultural University, Beijing, China; ^2^Key Laboratory of Viticulture and Enology, Ministry of Agriculture and Rural Affairs, Beijing, China

**Keywords:** Cabernet Sauvignon, rootstock, wine, flavonoids, color, OPLS-DA

## Abstract

Rootstocks are commonly utilized owing to their resistance to abiotic and biotic stress in viticulture. This study evaluated the effects of three rootstocks (1103P, SO4, and 5A) on the Cabernet Sauvignon (CS) vine growth, and their berries and wines flavonoids profiles in four consecutive vintages. The results showed that 1103P increased the pruning weight of CS and decreased the anthocyanin concentration in berries and wines, especially in the vintages with more rainy and cloudy days. 5A tended to decrease the pruning weight of CS and increase the anthocyanin concentration in berries and wines. Orthogonal partial least squares discriminant analysis (OPLS-DA) showed that the concentrations of total anthocyanins, F3’H-anthocyanins, malvidin-3-*O*-glucoside (Mv-glu), and malvidin-3-*O*-acetylglucoside (Mv-acglu) were the key substances affected by the rootstocks in CS berries and were significantly decreased by 1103P. Total anthocyanins, pinotins, Mv-glu, epicatechin, and vitisins were the rootstock-sensitive compounds that commonly differed in wines among the three comparison groups in the two vintages. Furthermore, 1103P brought more brightness to the wine and 5A gave the wine more red tones. In conclusion, rootstock 5A was recommended in the rainy and cloudy climate regions with regard to the berry flavonoids accumulation and the wine color.

## Introduction

Grape (*Vitis vinifera* L.) is one of the most important fruits in the world, and is grown in various climates (Delaunois et al., 2013). It is well known that winegrapes can achieve optimal maturity in a climate characterized by less rain and more sunshine during the growing season, such as in Mediterranean climates (Yu and Kurtural, 2020). A climate with excessive rain and inadequate light tend to limit the ripening of grapes. In such rainy and cloudy regions, proper viticultural practices are necessary to promote grape ripening and the accumulation of flavonoid compounds.

In viticulture, rootstock is a characteristic agronomic measure originating from phylloxera control. Phylloxera was once devastating to grape growth until the application of rootstocks saved the wine industry (Ollat et al., 2014). On the one hand, rootstocks also confer resistance to the scion, including resistance to salinity, drought, flooding and cold, which helps the vine survive adverse conditions (Suarez et al., 2019; Bianchi and Brancadoro, 2021). On the other hand, rootstocks will affect scion phenotypes, such as vine growth, yield, and berry physiochemical indices (Koundouras et al., 2009; Keller et al., 2012; Zhang et al., 2016; Chitarra et al., 2017; Nedelkovski et al., 2017; Romero et al., 2018; Cheng et al., 2020; Morales et al., 2021). Many research indicated that rootstocks with different characteristics confer different phenotypic traits on the scion. For example, rootstocks with high vigor increase yield and pruning weight, as well as the titratable acid content in the juice. Several studies, however, showed that rootstocks did not affect basic fruit composition metrics. For instance, Wang et al. (2019) found no significant differences in berry weight, pH and titratable acidity among the eight rootstock combinations of Cabernet Sauvignon. And Jogaiah et al. (2013) grafted Thomson saprophytic on 110 R and found 110 R did affect total soluble solids, acidity, and juice pH of Thompson Seedless. While, phenolic compounds, amino acids, and total proteins showed significant differences among rootstock-scion combinations. These inconsistent results regarding the rootstock effect may be due to cultural practices, varieties, and the different climatic conditions of the experimental sites, etc. For winegrapes, secondary metabolites tended to be more sensitive to the environment, so regions with high inter-annual climate change had more drastic changes in flavonoids and aromatic substances among years. And for plastic varieties, such as Cabernet Sauvignon, the metabolites could change greatly under different climatic conditions. A study showed that Cabernet Sauvignon tended to have contained higher levels of aldehydes, IBMP and *β-*damascenone under arid climatic conditions (Xu et al., 2015).

In winegrapes, flavonoids are important secondary metabolites, mainly anthocyanins, flavonols, and flavan-3-ols, which play a key role in wine coloration. Some researchers have found that rootstock affects flavonoid compound concentrations in berries. For example, Wang et al. (2019) revealed that 5A, Harmony, and Riparia Gloire enhanced the flavonol concentrations of Cabernet Sauvignon grapes, while SO4 slightly decreased most of the individual anthocyanin concentrations. A study reported that the Alicante Bouschet/1103P graft combination contained higher concentrations of total phenols and nonflavonoids, total monomeric anthocyanins, and monomeric and polymeric tannins in the seeds than own-rooted or other graft combinations (Titova et al., 2021). Nedelkovski et al. (2017) found a higher level of total phenols in the berries of Vranec grafted on Teleki rootstock, 41B could increase total anthocyanins concentrations in the berries, and Fercal could increase total flavan-3-ols concentrations in the berries. According to a recent study, 5BB was beneficial for the accumulation of total proanthocyanidins and various proanthocyanidin components in berry skins (Zhang et al., 2022). Together, these studies suggest that flavonoid compound concentrations in berries are mediated by rootstocks, and these differences are most likely due to an imbalance in vegetative and reproductive growth between rootstock combinations (Rasool et al., 2020).

Although extensive studies investigated how rootstocks affect the performance of scion varieties, it is still unclear whether rootstocks would make a difference in regions with much rain and little light during the grape growing season, especially during the berry ripening period. Additionally, there are limited reports on whether the differences in flavonoid concentration caused by rootstocks in grapes affect wines, specifically by changing the coloration of the wines produced. As mentioned above, grapes are grown in a wide range of climates, with many vines grown in areas with a rainy and low-light climate, such as the eastern, northeastern, and southern regions of China. The climate of the eastern China regions in the grape growing season is different from the mild and less rainy Mediterranean climate and is also different from the hot and dry climate of the western China regions. Given the combined influences among rootstock, cultivar, and environment, it is critical to conduct local long-term research to determine the type of rootstock to be utilized under specific edapho-climatic conditions (Ibacache et al., 2020). Therefore, we evaluated the rootstock in a specific region by several vintages to confirm the berry quality characteristics of different rootstock combinations. The effects of three rootstocks on the vine growth, berry and wine flavonoids, and color characteristics of Cabernet Sauvignon were evaluated to determine which specific compounds the rootstock would confer on the scion and identify suitable rootstocks for areas with less light and heavy rain.

## Materials and methods

### Experimental site and sampling

**Figure 1:**
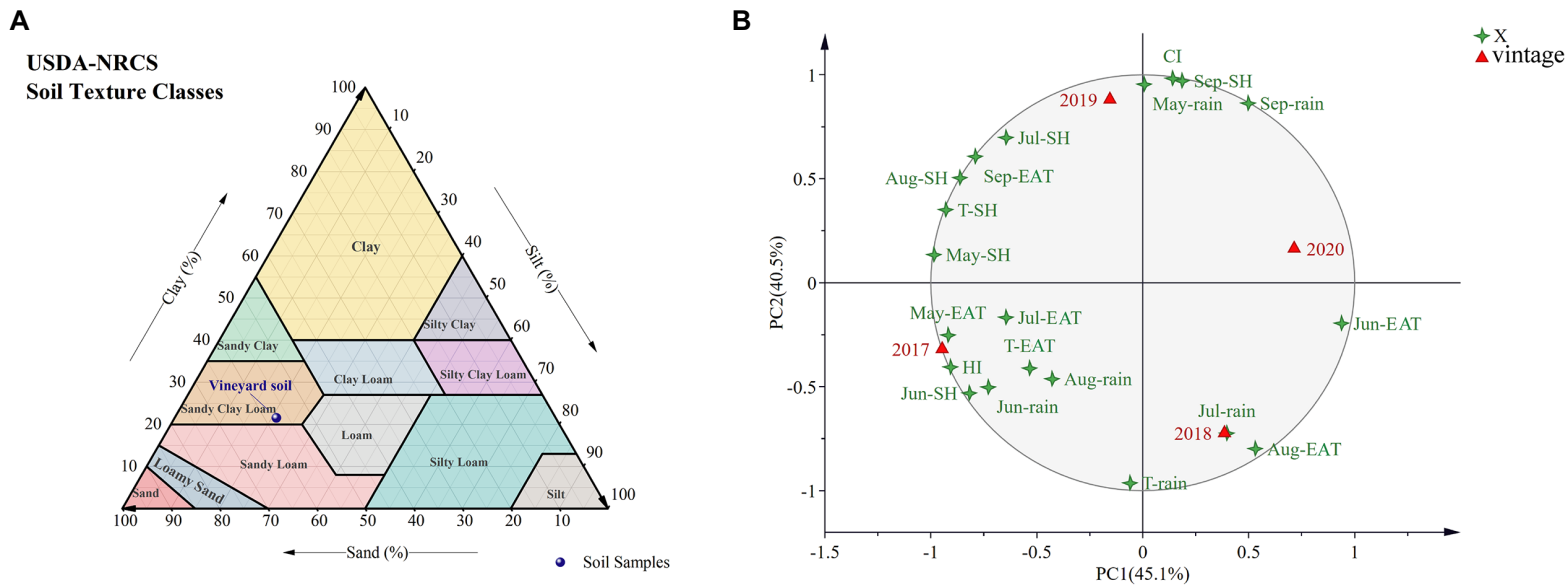
Texture of vineyard soil **(A)** and PCA based on meteorological data in 2017–2020 **(B)**.

The experiment was carried out at a Shangzhuang experimental station (40°14′N, 116°20′E, 49 m altitude) of China Agricultural University in Beijing, China, for four vintages (2017–2020). In this field experiment, *V. vinifera* L. cv. Cabernet Sauvignon (CS) clone 685 was grafted onto 1103P, SO4, and 5A (rootstock SSR maker information shown in Supplementary Table S1), Own-rooted CS was as the control. These vines were planted in 2012 and green-grafted in 2013, with rows oriented south to north, and row × vine spacing was 2.5 × 1.2 m. Furthermore, for the specific evaluation method according to Coombe (1995), E-L23 was the blooming period, E-L31 was berries pea-size, E-L35 was berries beginning to color and enlarge, E-L36 was berries with intermediate Brix values, E-L37 was berries not quite ripe, and E-L38 was berries harvested-ripe. Additionally, a modified vertical shoot positioning (M-VSP) training system was used in the vineyard (Cheng et al., 2014), and this system was spur-pruned and retained 12–15 nodes per linear meter. Each graft combination had three replicates, with each replicate containing 15 vines. Pest and nutrition control measures were implemented in line with local industry standards. The China Meteorological Data Interchange Platform^1^ provided the mean monthly temperature, sunshine hours, and rainfall during the grape growing season (Supplementary Table S2). In addition, the vineyard soils were classified as ‘sandy clay loam’ and contained 9.35 g/kg organic matter. Soil samples were taken at depths ranging from 20 to 60 cm and from 80 to 100 cm away from the vine’s side. Grapevine roots were mostly distributed between 20 and 60 cm deep in the vineyards studied. Nine sample plots were randomly chosen from the experimental vineyard, and each sample for each plot was taken at two different depths (20–40 cm, 40–60 cm). Then, nine sample points were randomly mixed into three replicates for analysis. Other soil features are listed in Figure 1A, Supplementary Table S3.

### Vine growth parameters, berry sampling, and winemaking

Leaf area, cluster weight, yield and pruning weight were determined in 2018–2020 according to Lu et al. (2022). The summer pruning weights (fresh weight) at different developmental stages (E-L 23, E-L 33, E-L 35.5, and E-L 37) were recorded during the growing season of 2020. At harvest, 300 berries were sampled and their physicochemical characteristics and flavonoid composition were determined. In addition, in 2019 and 2020, 20 kg of clusters of each replicate were manually crushed and placed in 20 l stainless steel containers for winemaking. After that, 0.6 g SO_2_ and 0.4 g pectinase (Optivin, Australia) were added to the must, and control the total SO_2_ content reached about 60 mg/l. Then pre-fermentation maceration was conducted at 18–20°C for 24 h, and 3.6 g commercial Lalvin strain D254 yeast (Laffort, France) was activated and inoculated into the must. In a temperature-controlled brewery workshop (23–25°C), alcoholic fermentation was carried out. The skins were punched down twice a day. When the reducing sugar level dropped below 1 g/l, the skins and seeds were then removed, and 0.02 g of *Lactobacillus* (Lalvin31, Lallemand Inc., French) was added to start the malolactic fermentation. When the malolactic fermentation ended, 1.2 g SO_2_ was added to the wines and control the total SO_2_ content reached about 80 mg/l. After that, the wines were bottled in 750 ml bottles and refrigerated in a wine cellar (12–16°C, without light) until analysis.

### Determination of berry physiochemical parameters

In each replicate, 100 berries were randomly selected for weighing and then squeezed into the must. The must was then centrifuged at 8,000 × *g* for 5 min to extract the supernatant. The juice’s total soluble solids were determined using a portable refractometer (PAL-1, Atago, Japan). A pH meter was used to determine the pH of the wine or juice (Sartorius PB-10, Germany). The titratable acidity of the juice was titrated with 0.05 mol/l NaOH to a pH 8.2 endpoint and represented as tartaric acid equivalents. The wine’s alcoholic degree, residual sugar, volatile acidity, and total acidity were measured according to the People’s Republic of China’s National Standard (GB/T15038-2006).

### Flavonoid compounds extraction and analysis

In a frozen state, berry skins were carefully peeled off. The skin was then pulverized into powder and freeze-dried at −40°C. Flavonols and anthocyanins were extracted following the procedure reported by Downey et al. (2007). The extraction of proanthocyanins was performed according to Liang et al. (2012) and Wang et al. (2019). Anthocyanins were analyzed with methods developed by our research group (Cheng et al., 2014). A diode array detector (DAD) and a reversed-phase Zorbax SB-C 18 column (250 × 4 mm, 5 μm) were employed on an Agilent 1,100 series HPLC-MSD trap VL (Agilent, Santa Clara, CA, United States). Flavonols were measured using an Agilent 1,200 series HPLC-MSD trap VL connected to a variable wavelength detector and a Zorbax EclipseXDB-C 18 column (250 × 4.6 mm, 5 μm). Flavan-3-ols in skins and seeds, as well as nonanthocyanin phenols in wines, were measured following the method described by Li et al. (2017). An Agilent 1,200 series HPLC system with a diode array detector (DAD) and a Poroshell 120 EC-C 18 column (1502.1 mm, 2.7 μm), coupled with Agilent 6,410 QqQ MS with an electrospray ionization source was employed. Anthocyanin concentrations were expressed as malvidin-3-*O*-glucoside equivalents, flavonol concentrations were expressed as quercetin-3-*O*-glucoside equivalents, flavan-3-ol concentrations were expressed as (+)-catechin (C), (−)-epicatechin(EC), (−)-epigallocatechin (EGC) and (−)-epicatechin-3-O-gallate (ECG) equivalents. All flavonoids from grapes were measured in mg/kg fresh weight (FW) and all phenols from wines were measured in mg/l wine.

### Determination of chromatic characteristics of wine

Wine chromatic parameters were measured with a Shimadzu UV-2450 UV–Visible spectrophotometer (Shimadzu Co., Kyoto, Japan) using 10 mm path length glass quartz cells and distilled water as a reference. The visible spectrum (400–700 nm) of wine was recorded at constant intervals (= 1 nm). Lightness (L*), red/green color coordinate (a*), yellow/blue color coordinate (b*), saturation (C_ab_*), and hue angle (H_ab_*) data were used to evaluate wine color using CIELab parameters (OIV, 2008). Each analysis was carried out three times.

### Statistical analyses

Analysis of variance (ANOVA) was performed using R 4.0.5 at *p* < 0.05 (Duncan’s multiple range test). The figures were drawn using OriginPro 2021 (OriginLab Corp, Northampton, MA, United States). Principal component analysis (PCA) and orthogonal partial least squares discriminant analysis (OPLS-DA) were carried out using SMICA14.1 (Umetrics, Umea, Sweden).

## Results and discussion

### Meteorological conditions

By analyzing the meteorological data for 4 years (Supplementary Table S2), it was found that 2017 was characterized by the longest sunshine hours; 2018 was characterized by the highest effective cumulative temperature and the most abundant rainfall; 2019 was characterized by the minimal rainfall; and 2020 was characterized by the shortest sunshine hours and lowest effective cumulative temperature indicating more cloudy days. Despite the lower rainfall in 2020, July/August rainfall accounted for 64% of the entire growing season. However, rainfall in 2019 accounted for only 48% of the total, and although 2017 had longer sunshine hours and effective cumulative temperature than 2019, rainfall was higher and more concentrated in July and August. A PCA was conducted based on 4 years of meteorological data (Figure 1B), and interestingly, the first principal component grouped 2017 and 2019 into one category, 2018 and 2020 into another category, the second principal component grouped 2019 and 2020 into one category, and 2018 and 2020 into another category. There are similarities in the climate in different years. The heliothermal index (HI) in 2017 was higher than that in other vintages, which indicated more light and heat in 2017, and the cool night index (CI) in 2019 was higher than in other vintages, which indicated more heat for the 2019 harvest season.

### Effects of rootstock on vine growth and phenological parameters

Rootstocks with different genetic backgrounds will confer different growth characteristics on scions (Table 1). Similar to previous reports, 1103P tended to increase scion pruning weights (Satisha et al., 2010; Keller et al., 2012) and was significantly different from 5A. And there was no significant difference between the CS/1103P combination and CS except at véraison (E-L35.5). The total pruning weights (E-L23 ~ E-L38) of CS/1103P at the five stages were significantly higher than those of CS; however, there were no consistent differences between CS/1103P and CS/SO4 (Supplementary Table S4). In addition, some studies have shown that 1103P improved the yield of scions (Di Filippo and Vila, 2011), and our results showed that 1103P had a tendency to improve yield in 2019, and showed significant differences in 2020. In general, previous studies have revealed that rootstock vigor and yield are positively correlated (Walker et al., 2002; Jones et al., 2009; Ollat et al., 2014). In contrast, in this study, rootstock 5A tended to weaken vine vigor. Although rootstock had a significant effect on the yield and pruning weight of the scion, it did not show a significant effect on Ravaz Index, which agreed with the findings of several previous studies (Koblet et al., 1995; Wang et al., 2019). Rootstock also did not have significant effects on leaf area and cluster weight. The influence of rootstocks on scion growth was mainly related to the genetic traits of different rootstocks. 1103P is a hybrid of *Vitlis berlandieri* and *V. rupestris*, and *V. rupestris* adapts to gravel and sandy soils with a deep rooting growth habit, while *V. berlandieri* is native to calcareous high pH soils (Zhang et al., 2016). From this, we inferred that 1103P with *V. rupestris* and *V. berlandieri* as parents absorbed more water and nutrients to supply the vegetative growth of the scion, resulting in a higher pruning weight of the scion. 5A and SO4 are hybrids of *V. berlandieri* and *V. riparia*, with shallow rooting growth habit and adapted to relatively wet environments. Although 5A and SO4 had the same parentage, SO4 conferred a higher scion biomass, which showed that even rootstocks with the same parentage still have different effects on the scion.

**Table 1:** Effects of rootstock on vine growth parameters.

Vintage	Graft combination	Cluster weight (g)	Leaf area/vine (m^2^)	Leaf area/yield (m^2^/kg)	Yield (kg/vine)	Pruning weight (kg/vine)	Ravaz index kg (yield)/kg (pruning weight)
2018	CS	122.57 ± 5.34	7.47 ± 0.78	2.03 ± 0.15	3.68 ± 0.16	2.01 ± 0.15ab	1.84 ± 0.17
	CS/1103P	120.89 ± 3.45	8.06 ± 0.67	2.19 ± 0.28	3.71 ± 0.18	2.28 ± 0.19a	1.64 ± 0.18
	CS/5A	118.64 ± 4.05	7.14 ± 0.52	2.01 ± 0.19	3.56 ± 0.12	1.86 ± 0.14b	1.91 ± 0.10
	CS/SO4	125.65 ± 4.62	7.70 ± 0.42	2.04 ± 0.14	3.77 ± 0.14	2.13 ± 0.24ab	1.79 ± 0.28
2019	CS	127.54 ± 2.11	6.18 ± 0.54	1.61 ± 0.19	3.28 ± 0.22	1.71 ± 0.18ab	2.02 ± 0.28
	CS/1103P	124.36 ± 5.10	6.85 ± 0.53	1.70 ± 0.15	3.42 ± 0.24	2.00 ± 0.22a	1.72 ± 0.27
	CS/5A	122.52 ± 5.16	5.95 ± 0.95	1.54 ± 0.17	3.25 ± 0.24	1.64 ± 0.13b	1.91 ± 0.25
	CS/SO4	125.29 ± 4.82	6.64 ± 0.61	1.68 ± 0.10	3.36 ± 0.14	1.70 ± 0.15ab	1.99 ± 0.11
2020	CS	126.99 ± 5.18	6.77 ± 0.76	2.05 ± 0.19	3.37 ± 0.21b	1.96 ± 0.24ab	1.69 ± 0.15
	CS/1103P	131.26 ± 5.34	7.54 ± 0.54	1.90 ± 0.19	3.98 ± 0.31a	2.30 ± 0.32a	1.74 ± 0.24
	CS/5A	121.01 ± 7.79	6.63 ± 0.65	2.03 ± 0.09	3.33 ± 0.24b	1.78 ± 0.26b	1.87 ± 0.32
	CS/SO4	130.26 ± 5.45	7.01 ± 0.88	2.07 ± 0.31	3.45 ± 0.25b	2.00 ± 0.21ab	1.70 ± 0.16
Rootstock		ns	ns	ns	*	*	ns
Vintage		*	ns	ns	*	ns	ns
Rootstock × vintage		ns	ns	ns	*	ns	ns

Data were reported as mean ± standard deviation (*n* = 3). Different letters in the same column within the same year manifest significant differences according to Duncan’s test (*p* < 0.05). Two-way ANOVA tests for significance levels of vintage, rootstock, and rootstock × vintage differences (“ns”, not significant; *p < 0.05; **p < 0.01; ***p < 0.001).

In addition, three consecutive years (2018–2020) of phenological periods showed that 1103P delayed the grape development process (Supplementary Table S5). There was no significant difference in flowering time between the three graft combinations and CS. In E-L23, CS/SO4 and CS/5A did not show differences with CS, while CS/1103P was 1–2 days later than CS. In E-L35, CS/5A was 1–2 days earlier than CS, CS/SO4 was 1–2 days earlier than CS in 2018 and 2019, and CS/1103P was 2–3 days later than CS. In E-L36, CS/5A was 1 day earlier than CS, CS/SO4 was 3 days later than CS (2018 and 2020), and there was no difference in 2019, and CS/1103P was 4–5 days later than CS. In E-L37, CS/5A was 1 day earlier than CS, CS/SO4 was 2–3 days later than CS, and CS/1103P was 4–5 days later than CS. As development progressed, the phenological stages of different graft combinations gradually diverged, and the maximum gap was reached at the E-L36 stage, after which there was a tendency for the difference in phenological period among rootstock combinations to decrease. This suggested that 1103P tended to delay berry ripening, especially during the véraison period. It is worth noting that the later the véraison period started, the greater the number of days required to complete the coloration. Furthermore, the vintage affected the length of the phenological period. Three graft combinations and CS had the shortest véraison period in 2019 compared with other vintages. In 2020, these vines had a longer véraison period. For CS/1103P combination, it was longer than 6 days in 2019. We speculated that the vintage with sufficient light (2019) could mask the differences brought by rootstocks and that the vintage with insufficient light (2020) further delayed berry development. Azuma et al. (2019) found that sufficient light promotes grape fruit ripening.

### Effects of rootstock on berry and wine physicochemical parameters

Over four consecutive vintages, the rootstocks did not affect the weight of the berries (Table 2), which was consistent with some prior observations (Wooldridge et al., 2010; Berdeja et al., 2014; Wang et al., 2019). In terms of total soluble solids in berries, the CS/5A combination had a higher content than CS/1103P, and did not show consistent higher TSS content than CS/SO4, although there was no significant difference when compared to CS. Rootstocks also had an impact on the titratable acidity and pH of juice. In this study, 1103P significantly improved the juice titratable acidity in two vintages (2019, 2020), but it did not show any significant differences with other combinations in 2017 and 2018 and performed the same with CS/SO4. For the pH of the juice, there was a small difference among the three graft combinations and the own-rooted CS. Two-way ANOVA showed a combined influence between vintages and rootstocks, with high soluble solids and low titratable acidity in berries in vintages with more light and less rain, such as 2019. This could be because adequate light and less precipitation promote the accumulation of soluble solids and accelerate grape ripening(Keller, 2010; Gutiérrez-Gamboa et al., 2021). For the wine physicochemical properties, there was no significant difference between the three combinations and the own-rooted CS. Overall, we found that alcohol content and residual sugar in 2019 were higher than that in 2020. and the total acid content in 2020 was higher than that in 2019.

**Table 2:** Physicochemical parameters of graft combinations in berries and wines.

Vintage	Graft combination	Grape				Wine				Total acidity (g/l)
		Berries weight (g/100 berries)	TSS (°Brix)	Titratable acidity (g/l)	pH	Alcohol (%, *v*/*v*)	pH	Residual sugar (g/l)	Volatile acidity (g/l)	
2017	CS	155.70 ± 4.50	20.25 ± 0.49a	6.17 ± 0.19ab	3.09 ± 0.02	NA	NA	NA	NA	NA
	CS/1103P	148.03 ± 3.92	18.80 ± 1.41b	6.88 ± 0.59ab	2.98 ± 0.04	NA	NA	NA	NA	NA
	CS/5A	149.14 ± 2.46	20.30 ± 0.14a	5.57 ± 0.09b	2.97 ± 0.02	NA	NA	NA	NA	NA
	CS/SO4	147.93 ± 3.30	19.05 ± 0.92ab	7.46 ± 0.23a	3.05 ± 0.07	NA	NA	NA	NA	NA
2018	CS	130.00 ± 3.45	21.27 ± 0.12a	9.63 ± 0.22a	3.41 ± 0.04b	NA	NA	NA	NA	NA
	CS/1103P	130.67 ± 5.13a	19.80 ± 0.30b	10.75 ± 0.22a	3.44 ± 0.02ab	NA	NA	NA	NA	NA
	CS/5A	124.7 ± 5.51ab	21.23 ± 0.10a	7.87 ± 0.37b	3.47 ± 0.01a	NA	NA	NA	NA	NA
	CS/SO4	129.33 ± 7.09a	20.30 ± 0.20b	10.50 ± 1.72a	3.33 ± 0.01c	NA	NA	NA	NA	NA
2019	CS	141.57 ± 10.53a	21.50 ± 0.21a	7.81 ± 0.09c	3.47 ± 0.04a	11.20 ± 0.20	3.69 ± 0.03	0.84 ± 0.09	0.45 ± 0.05	7.10 ± 0.26
	CS/1103P	138.81 ± 5.36ab	20.40 ± 0.26b	8.91 ± 0.01a	3.43 ± 0.02ab	11.10 ± 0.12	3.70 ± 0.04	0.75 ± 0.15	0.47 ± 0.03	7.30 ± 0.20
	CS/5A	154.92 ± 4.39a	21.37 ± 0.46a	7.92 ± 0.01bc	3.40 ± 0.02ab	11.20 ± 0.15	3.68 ± 0.02	0.86 ± 0.11	0.49 ± 0.02	7.13 ± 0.32
	CS/SO4	146.91 ± 9.52a	20.73 ± 0.21b	8.80 ± 0.01ab	3.41 ± 0.03ab	11.07 ± 0.15	3.67 ± 0.02	0.77 ± 0.04	0.43 ± 0.04	7.40 ± 0.26
2020	CS	153.17 ± 5.75a	20.37 ± 0.37a	7.92 ± 0.33b	3.43 ± 0.01a	10.70 ± 0.10	3.69 ± 0.03	0.68 ± 0.08	0.41 ± 0.03	7.43 ± 0.31
	CS/1103P	147.63 ± 8.85ab	18.47 ± 0.23b	8.80 ± 0.19a	3.25 ± 0.09b	10.43 ± 0.21	3.70 ± 0.04	0.59 ± 0.10	0.40 ± 0.03	7.73 ± 0.25
	CS/5A	145.43 ± 3.85a	19.87 ± 0.85a	7.81 ± 0.70b	3.45 ± 0.01a	10.50 ± 0.20	3.68 ± 0.02	0.62 ± 0.05	0.39 ± 0.04	7.53 ± 0.15
	CS/SO4	149.40 ± 6.61a	19.10 ± 0.17ab	8.91 ± 0.57a	3.38 ± 0.02ab	10.47 ± 0.15	3.67 ± 0.02	0.69 ± 0.03	0.42 ± 0.05	7.67 ± 0.21
Rootstock		ns	*	*	ns	ns	ns	ns	ns	ns
Vintage		ns	**	**	*	**	ns	**	ns	**
Rootstock × vintage		ns	ns	ns	ns	ns	ns	ns	ns	ns

Data were reported as mean ± standard deviation (*n* = 3). Different letters in the same column within the same year manifest significant differences according to Duncan’s test (*p* < 0.05). Two-way ANOVA tests for significance levels of vintage, rootstock, and rootstock × vintage differences (“ns”, not significant; *p < 0.05; **p < 0.01; ***p < 0.001).

### Flavonoid compositions in berries

#### Effects of rootstocks on anthocyanin concentration in berries

In agreement with some researchers (Ozden et al., 2010; Gutiérrez-Gamboa et al., 2019), different rootstocks significantly affected anthocyanin concentrations in berries, especially in the growing season with more rain and less light. 1103P reduced the total anthocyanin concentration in berries compared with the own-rooted berries in four consecutive vintages. SO4 tended to attenuate the anthocyanin concentrations in 2017 and 2020 but was still higher than that in the CS/1103P combination. 5A did not show a significant difference compared to CS (Supplementary Table S6). In this study, 16 kinds of anthocyanins were detected in skins, and malvidin was the most dominant type of anthocyanin in the skins. 1103P grafted Cabernet Sauvignon had a significantly lower concentration of malvidin derivatives than CS, regardless of the vintage, and this difference was more pronounced in 2020. One interpretation of this finding is that the rootstock characteristics are more pronounced in a stressful environment, while vintages with less rain and more light tended to mask this difference. Excessive rainfall and insufficient light would limit anthocyanin accumulation no matter what variety/rootstock combination was used. Additionally, CS/1103P also showed lower 3′5′-hydroxylated anthocyanin (F3′5′H) concentrations in 2017 and 2020. It is well known that 3′-hydroxylated (F3′H) and 3′5’-hydroxylated (F3′5′H) pathways are two branches of flavonoid metabolism. 1103P mainly decreased the anthocyanin concentrations of F3′5′H, and the anthocyanin concentrations of F3′H showed a relatively small difference. For different acylated types of anthocyanins, coumarylated and acetylated anthocyanins were likewise dramatically decreased by 1103P. 1103P had the highest vine vigor (pruning weight) and tended to increase leaf area, causing depression of the leaf canopy of the vines and blocking light, thus affecting the accumulation and synthesis of anthocyanin (Lu et al., 2021). In addition, excessive pruning weight meant that more photosynthetic products synthesized by the leaves were accumulated in the branches (Bascuñán-Godoy et al., 2017), changing the source-sink relationship, which might also explain the lower sugar content of CS/1103P combinations. As previously reported, 1103P caused a decrease in the concentration of anthocyanin in berries, and the ripening rate of Cabernet Sauvignon grape berries was observed to be delayed when grafted onto 1103P (Corso et al., 2016). The CS/1103P combination had higher reproductive growth than CS, which might vary the amount of sunlight taken by grape berries and inhibit reproduction, which could explain the reductions in anthocyanins caused by 1103P. There was a report that the expression of the genes encoding key enzymes in the anthocyanin biosynthesis pathway was regulated by sugars (Gollop et al., 2001). As a result, reduced anthocyanin concentrations may have resulted from the lower sugar content of CS/1103P compared to CS. Furthermore, SO4 decreased the anthocyanin concentrations in Cabernet Sauvignon skins, as similar to Wang et al. (2019). And the CS/SO4 combination had a higher anthocyanin concentration than the CS/1103P combination, Ozden et al. (2010) also found similar results in Shiraz under different watering management approaches. CS/SO4 had more sugars than CS/1103P, which may explain why the CS/SO4 combination had higher anthocyanin concentrations. CS/5A combination had higher sugar content and anthocyanin concentrations compared with CS/1103P and CS/SO4, although it did not show a significant difference with CS. Wang et al. (2019) also found the CS/5A combination had higher anthocyanin concentrations compared with the CS/SO4.

For all graft combinations, the concentrations of anthocyanins in berries were higher in 2019 than in other vintages. From the meteorological data, we found that the vintage had less rain and enough light, especially during the véraison period, which was beneficial for the synthesis of anthocyanins. In such vintage, the rootstock had less effect on the concentration of anthocyanins in the scion, while in a vintage with insufficient light and more rainfall, the rootstock had a greater effect on the total concentration of anthocyanins in the scion. The percentage change of total anthocyanin concentrations for the same graft combination differed among vintages. In 2019, the CS/1103P combination only decreased the total anthocyanin concentrations by 8.04% compared with CS, while in 2020, the anthocyanin concentrations of the CS/1103P combination decreased by 24.93% compared to CS. This demonstrated that a stressful environment with less light and more rain increased the effect of rootstocks on berry anthocyanin concentrations and the proper use of rootstock was significant for the scion to adapt to the stressful survival environment. Oliveira et al. (2020) used IAC 572 and 1103P to graft Alicante Bouschet, and it was found that rootstock and year had a synergy effect, and year had a greater effect on flavonoid compound concentrations. A similar discovery was made on Syrah. (Dias et al., 2017). In these studies, the anthocyanin concentrations of the same treatment among different vintages were much greater than that of different treatments of the same vintage, so we considered that vintage was the determining factor of anthocyanin concentrations in berries.

#### Effects of rootstocks on flavonol concentrations in berries

In our study, it was found that the CS/1103P combination had a lower flavonol concentration in skins in all vintage, this decrease was mainly caused by the low concentrations of quercetins and myricetins, and the CS/5A combination had higher flavonols in most vintages. In addition, the CS/SO4 combination did not show a significant difference compared with CS (Supplementary Table S6). A study found that 5A, Harmony, and Riparia Gloire increased the concentrations of flavonols in Cabernet Sauvignon in M-VSP (Wang et al., 2019). Obviously, the vintages were key in determining flavonol concentrations. In 2017 and 2019, flavonols showed higher levels compared with other vintages. Many studies have shown that the response to light treatments substantially increased flavonol concentrations due to the increased expression of key genes including flavonol synthase (Matus et al., 2009; Sternad Lemut et al., 2011; Feng et al., 2015). There was a higher flavonol concentration in vintages with less rain and more sunshine, especially in 2019. In four vintages, the CS/5A combination had a higher flavonol concentration than the CS/1103P combination. This difference was mainly caused by quercetins, suggesting that rootstock 5A was likely to favor the synthesis of quercetins, while 1103P was unfavorable to the synthesis of quercetins, as Table 1 shows, 1103P had a higher pruning weight, which caused branch depression and resulted in insufficient light, thus affecting the accumulation of flavonols, while 5A had a relatively low pruning wight, which allowed the grapevines to receive more light, thus favoring the synthesis of flavonols. Quercetins were mainly produced by the F3′H pathways, and myricetins was mainly produced by the F3′5H′ pathways, suggesting light might have a greater effect on flavonols synthesized by F3′H pathways than by F3′5H′ pathways. A study had shown temperature, light exposure and vine water stress would effect on the expression of genes encoding F3′H and F3′5H′ in grapes (Robinson et al., 2019).

#### Effects of rootstocks on flavan-3-ol concentrations in berries

Flavan-3-ols can be found in many tissues, most of them accumulate in the grape berry seeds (Montealegre et al., 2006). The rootstock did not have a significant effect on the flavan-3-ol concentrations, suggesting that the flavan-3-ol concentration was relatively stable and not susceptible to the effects of cultivation practices. Although there was no significant difference in flavan-3-ol concentrations between rootstock combinations, 1103P still tended to promote flavan-3-ol accumulation and 5A tended to reduce flavan-3-ols accumulation (Supplementary Table S6). This might be the low TSS at harvest (around 21°Brix), which could have resulted with more condensed tannin accumulation. If berries reached higher ripening stages, the results might change since tannins will decrease in amounts or “softened” by polymerizing with other flavonoids. Unlike anthocyanins, flavan-3-ols were lower in a vintage (2019) with sufficient light and less rain, and higher in a vintage (2020) with insufficient light and more rain. This difference might be due to the accumulation pattern of flavan-3-ols. Studies have shown that the accumulation pattern of flavan-3-ols in grape berries exhibited a trend of increasing and then decreasing (Padilla-González et al., 2022), generally reaching the highest concentration at the véraison stage, and then showing a decreasing trend, but the synthesis of anthocyanin showed a rapid accumulation process during the véraison stage. In combination with the phenological period, vintages with insufficient light often have more rainfall during the véraison stage, and rainfall slows down the accumulation of anthocyanin and flavonol, while the decomposition rate of flavan-3-ol decreases, which in turn causes the phenomenon of low anthocyanins and flavonols concentrations and high flavan-3-ols concentrations in berries at maturity in vintages with insufficient light and more rainfall (Blancquaert et al., 2019).

According to previous studies, rootstocks did not significantly change flavan-3-ol concentrations in grape berries (Koundouras et al., 2009; Harbertson and Keller, 2012; Wang et al., 2019). In our study, the results also showed that rootstock had no significant effects on flavan-3-ol concentrations in skins and seeds, although there was some difference between flavan-3-ol monomers, which was consistent with previous reports. Consistent with the majority of studies, the vintages were still the main reason for the difference in flavan-3-ol concentrations in grape skins among grafted and own-rooted vines. Merlot grapevines grafted onto the 1103P and SO4 rootstocks all had increased the flavan-3-ol concentration to some degree (Gutiérrez-Gamboa et al., 2019). In comparison to the other rootstocks, grapevines grafted onto SO4 had a higher concentration of total proanthocyanidins in skins and seeds (Gutiérrez-Gamboa et al., 2019).

### Effects of rootstocks on wine flavonoid compounds

Anthocyanins are major color-contributing compounds in red wine, and the concentration and percentage of different anthocyanin derivatives determine the color quality (Wang et al., 2019). In this study, 16 anthocyanin compounds were detected in wines for two consecutive years. The total anthocyanin concentration of the wines in 2019 was significantly higher than that of the wines in 2020. This was mainly due to more light and less rain in 2019, a climate condition that favors the accumulation of anthocyanin in grapes, which could be a richer substrate for the wine (Blancquaert et al., 2019). And CS/1103P combination had lower total anthocyanin concentration in the wine compared with CS and other graft combinations in 2020, and there was little difference among the three graft combinations and CS in 2019 (Supplementary Table S7). This also seemed to confirm that in vintage (2019) with sufficient light and little rain the differences brought by the rootstock were masked, while in vintage (2020) with little light and lots of rain the specificity of the rootstock could only be fully revealed. The CS/5A combination had higher anthocyanin concentrations in the wine, although there was no significant difference between CS and CS/5A. Notably, this trend was consistent with the anthocyanin concentrations in the berries. CS/1103P included the lowest concentration of malvidin-3-glucoside. The same trend was observed in the CS/1103P combination wines. For Merlot, wines from grapevines grafted onto SO4, 140R, Gravesac, and 4,453 M rootstocks presented better chromatic characteristics and a higher anthocyanin concentration than 1103P, 99R, 101–14,110R (Gutiérrez-Gamboa et al., 2019). However, a recent study showed that the Alicante Bouschet/1103P rootstock combination contained higher concentrations of total monomeric anthocyanins than the Alicante Bouschet/IAC 572 rootstock combination in a tropical semiarid climate. Furthermore, the rootstock showed a significant impact on Pinot noir wine tannin levels and its extraction rate under moderate climatic conditions, Scions grafted onto SO4, were characterized by a 15% higher tannin concentration in berry seed and skin compared to those grafted onto the Riparia Gloire de Montpellier, while final tannin concentration in wines depended on the rootstock (Blank et al., 2022). A study showed 110R improved total phenol and anthocyanin content in Monastrell wines (Navarro et al., 2021). But for Carignan grapevines in the Maule Valley, rootstock barely affected phenolic content in wines (Gutiérrez-Gamboa et al., 2018). These studies showed that rootstocks did affect the flavonoid concentration of wines, but that rootstocks did not have a consistent effect on the flavonoid content of scions. This difference might be attributed to differences in the environment, cultural practices and scion (Oliveira et al., 2020). In our study, 1103P reduced the anthocyanin concentrations in wines, while 5A did not significantly affect the anthocyanin concentration of wine, consistent with the trend in grapes, suggesting that rootstocks could indeed affect anthocyanin concentrations in wine by altering anthocyanin concentrations in grapes.

Anthocyanin derivatives are also key components in the color of red wines, and they are crucial in preserving color stability during aging (Zhang et al., 2021). In this research, five types of anthocyanin derivatives were determined in two vintages, including Pinotins, Vitisins, Flavanyl-pyranoanthocyanins (A-v-F), and Anthocyanin ethyl-linked flavan-3-ols, which are direct anthocyanin-flavanols condensation products(A-F/F-A). As we expected, vintage was still a significant factor in determining the concentration of anthocyanin derivatives. The anthocyanin derivatives in 2020 wines were much higher than in 2019 wines. With more rain and less light in 2020, the berries contained more flavan-3-ols, which contained more flavan-3-ols and may have provided more substrates for the formation of the anthocyanin derivatives. However, there was no significant difference among the four graft combinations in 2019. In addition, a total of 12 kinds of flavonols were detected in wines in the 2 vintages, including 5 kinds of quercetins, 3 kinds of myricetins, 2 kinds of kaempferols, and 1 kind of isorhamnetin (Supplementary Table S7). In 2019, the rootstock did not affect flavonol concentrations in Cabernet Sauvignon wines, however, in 2020, flavonol concentrations in wines from different rootstock combinations exhibited substantial variations. The maximum flavonol level was found in the CS/5A combination, whereas the lowest flavonol concentration was found in the CS/1103P combination, which was due to the different levels of quercetin in the wines. Besides, seven kinds of flavan-3-ols were detected in wines in the two vintages, including catechin, epicatechin, epigallocatechin, gallocatechin, and procyanin B1, procyanin B2, and procyanin C1. Consistent with the trend in flavan-3-ol concentration in berries, the flavan-3-ol concentration in the wine of different rootstock combinations in the 2 vintages also did not show significant differences. Individual substances showed differences in 2020, such as catechin, gallocatechin, and procyanin B1. Wines in 2020 contained a higher concentration of flavan-3-ols, maybe due to the rainy and low light conditions of the 2020 vintage, which slowed down the breakdown of proanthocyanidins in the later development stages of grapes (Blancquaert et al., 2019), and this difference was further reflected in the wines.

The above analysis revealed that different rootstocks had little effect on anthocyanins, anthocyanin derivatives, and nonanthocyanin phenols in Cabernet Sauvignon wines in 2019, but showed there were significant differences in 2020. The effect of the rootstock on the scion was more pronounced in vintages with high environmental stress (more rainy and cloudy days) and did not show differences in years with low stress, which was consistent with our perception that rootstocks are resistant, and this fully illustrates the need to use rootstocks in stressful environments.

### Principal component analysis and orthogonal partial least squares discriminant analysis of compounds in berries and wine

**Figure 2:**
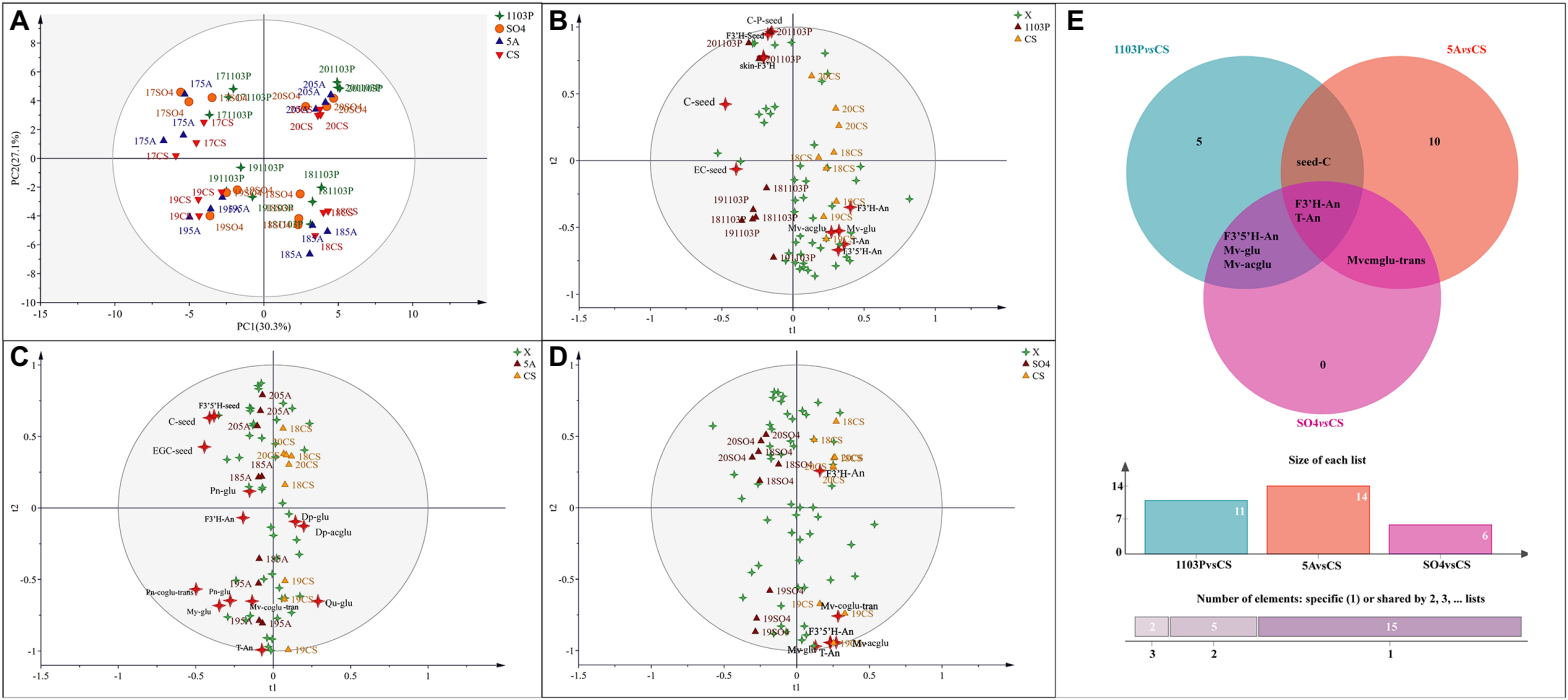
Principal component analysis (PCA) based on flavonoid compound concentrations of grapes in four vintages (2017–2020) **(A)**; Orthogonal partial least squares discriminant analysis (OPLS-DA) based on flavonoid compound concentrations of grapes in three vintages (2018–2020) **(B–D)**; Venn diagram based on differential compounds (VIP > 1) in grapes **(E)**. The abbreviated names of the substances in the figure are shown in Supplementary Table S6.

In grapes, to further identify the characteristics of flavonoid metabolites in different graft combinations, PCA was used for analysis. Regrettably, PCA did not separate the rootstock combinations but rather separated them from vintages to vintages, suggesting that vintage played a key role in flavonoid concentrations in berries (Figure 2A). The first principal component (PC1) explained 30.3% of the total variance, classifying 2017 and 2019 into one group and 2018 and 2020 into another. As shown in Figure 1B, the climatic conditions were similar in 2017 and 2019, and in 2018 and 2020. We speculated that similar vintages can confer similar compound components to the berries. The second principal component (PC2) explained 27.1% of the total variance, classifying 2017 and 2020 into one group and 2018 and 2019 into another. To reduce the interference between vintages, OPLS-DA was used in different graft combinations. However, when we tried to train the model with all vintages to uncover further differences between the different rootstock combinations and the own-rooted vine, the model was unreliable, even though we could discriminate between grafted combination and own-rooted very well. We were able to construct a more reliable OPLS-DA model by removing the flavonoids in 2017 and fitting them with other vintages. As shown in Figure 2B, the first principal component distinguished the CS/1103P combination and CS, and the second principal component distinguished the vintages. By screening compounds with VIP >1, we could see that T-An, F3′5′H-An, F3′H-An, Mv-glu, Mv-acglu, C-seed, skin-F3′H, Seed-F3′H, and C-P-seed were the compounds that distinguished CS/1103P combination from CS. Similarly, we established the OPLS-DA model for the CS and SO4/CS combination (Figure 2D), and Mv-acglu, Mv-coglu-trans, Mv-glu, T-An, and F3’H-An can be regarded as the differential compounds to distinguish them. For the CS and CS/5A combination (Figure 2C), Mv-coglu-trans, Dp-acglu, Pn-coglu-trans, Dp-glu, F3′H-An, Qu-glu, EGC-seed, C-seed, and F3′5′H-seed could be used as the differential compounds to distinguish them. Overall, anthocyanins were the main metabolites affected by rootstocks in the rainy area, especially malvidin-based anthocyanin. In addition, the Venn diagram was used to further search for the common differential compounds in the three groups (Figure 2E). We found that T-An and F3′H-An were the common differential compounds among the three groups, showing rootstock did affect anthocyanin concentration in berries to some degree.

**Figure 3:**
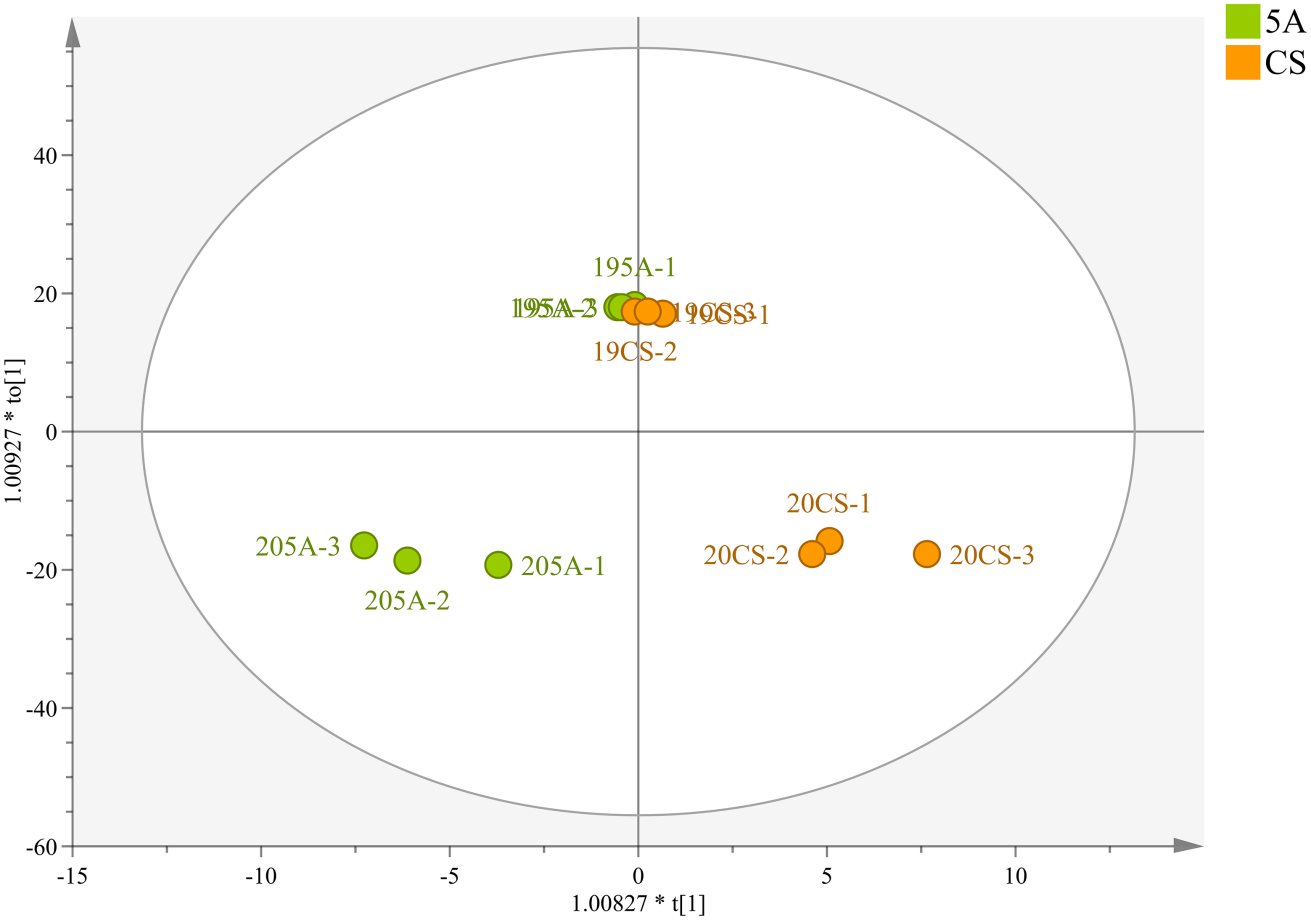
OPLS-DA based on flavonoid compound concentrations in wine of CS and CS/5A in the 2019 and 2020 growing seasons.

**Figure 4:**
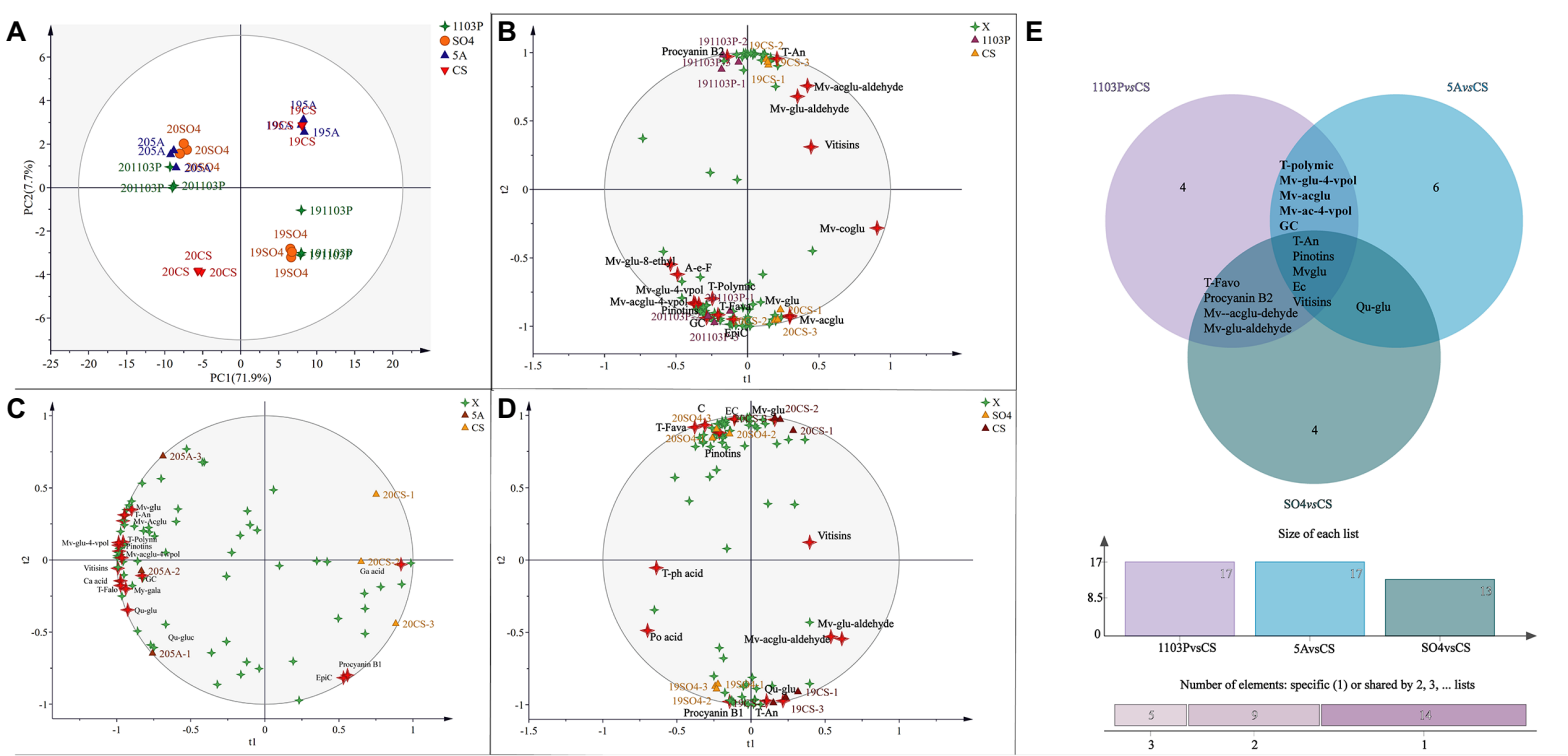
Principal component analysis (PCA) based on flavonoid compound concentrations of wine in two vintages (2019–2020) **(A)**; OPLS-DA based on flavonoid compound concentrations of wine in two vintages (2019–2020) **(B–D)**; Venn diagram based on differential compounds (VIP > 1) in wines **(E)**. The abbreviated names of the substances in the figure are shown in Supplementary Table S7.

Principal component analysis (PCA) was also used to classify the different graft combinations for wines in two vintages. As shown in Figure 4A, the first two principal components explained 79.6% of the total variance. PC1 accounted for 71.9% of the total variance, which could separate the two vintages. Wine in 2019 included more malvidins, quercetins, and kaempferol (Supplementary Table S7), whereas the anthocyanin derivatives in 2020 were higher than those in 2019, which was mainly because wines in 2020 contain more proanthocyanidin, providing more substrates for the formation of anthocyanin derivatives. In addition, OPLS-DA was used again to search for differential compounds imparted to wines by different rootstock combinations. For the CS/1103P and CS/SO4 combinations, the 2-vintage data could be well separated, but for the CS/5A combination, the 2019 compound concentrations were very similar to CS and there was no way to distinguish them (Figure 3), so we fitted the OPLS-DA model using only compounds in 2020, and the results could well distinguish CS/5A from CS. The differential compounds are shown in Figures 4B–D. By screening the compounds with VIP > 1, it could be seen that there were 17 kinds of differential compounds between the CS/1103P combination and CS, 17 kinds of differential compounds between the CS/5A combination and CS, and 13 kinds of differential compounds between the CS/SO4 combination and CS. The intersection of the three groups of differential compounds was taken and it was found that T-An, Mv-glu, pinotins, vitisins, and EC were the differential compounds common to all three graft combinations. This indicated that these substances were the key substances of the rootstock influencing wine compounds.

### Partial least squares regression screening of key flavonoid compounds affecting wine colorimetric parameters

Wine colorimetric parameters were measured to better analyze the effect of differences in substance concentrations on wine color in rootstock combinations (Table 3). The colorimetric parameters of the two vintages together demonstrated that the CS/1103P produced wines with higher L* and H_ab_*, which indicated that CS/1103P wine is brighter. And the CS/5A had a higher a* and *C*_ab_* values, meaning the CS/5A wines had more of a red hue. Compared to the 2 vintages, the 2019 wines had significantly higher a*, and *C*_ab_* values than 2020 wines and significantly lower L* and H* values than 2020.

**Table 3:** Colorimetric parameters of Cabernet Sauvignon wines (clone 685) and three graft combinations (CS/1103P, CS/SO4, and CS/5A) during two growing seasons (2019–2020).

Vintage	Graft combination	a*	C_ab_*	H_ab_*	L*	b*
2019	CS/1103P	15.65 ± 1.08c	15.98 ± 1.23c	10.18 ± 1.62a	84.2 ± 2.71a	3.13 ± 1.12ab
	CS/5A	23.02 ± 0.40a	23.18 ± 0.41a	6.62 ± 0.31b	80.34 ± 0.29b	2.67 ± 0.16b
	CS	23.76 ± 0.08a	24.13 ± 0.10a	10.09 ± 0.55a	78.62 ± 0.89b	4.23 ± 0.24a
	CS/SO4	20.19 ± 0.29b	20.4 ± 0.31b	8.25 ± 0.49ab	81.12 ± 1.34b	2.93 ± 0.21b
2020	CS/1103P	8.03 ± 0.31c	8.72 ± 0.50c	22.66 ± 3.53a	92.12 ± 0.46a	3.37 ± 0.67ab
	CS/5A	10.93 ± 0.12b	11.41 ± 0.10b	16.53 ± 3.35a	89.55 ± 0.47b	3.25 ± 0.66b
	CS	10.98 ± 0.30b	12.03 ± 0.53ab	23.95 ± 2.16a	88.92 ± 0.59bc	4.89 ± 0.64a
	CS/SO4	11.84 ± 0.62a	12.55 ± 0.45a	19.00 ± 5.35a	88.25 ± 0.21c	4.07 ± 1.08ab

Data were reported as mean ± standard deviation (*n* = 3). Different letters in the same column within the same year manifest significant differences according to Duncan’s test (*p* < 0.05). “ns” indicates not significant (*p* > 0.05).

**Figure 5:**
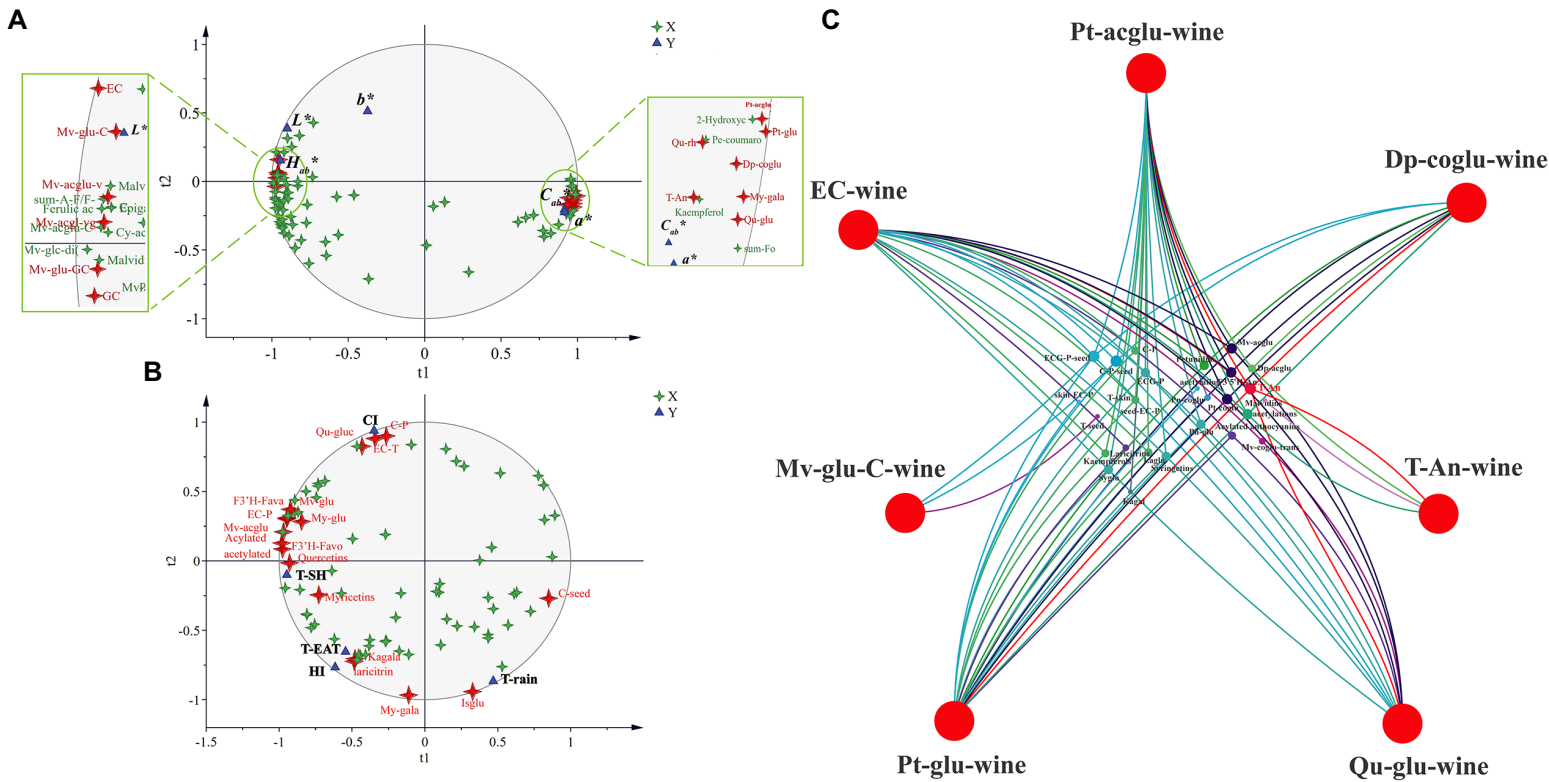
Partial least squares regression (PLSR) analysis for all wines based on CIELAB parameters and flavonoid compounds **(A)**; PSLR analysis for grapes based on flavonoid compounds and environmental factors **(B)**; PSLR analysis for all wines based on key flavonoid compounds affecting wine color parameters and flavonoids in grapes **(C)**. The abbreviated names of the substances in the figure are shown in Supplementary Tables S6, S7.

In addition, partial least squares regression (PLSR) analysis revealed that Pt-aclgu, Pt-glu, My-gala, Qu-glu, Qu-rh, and T-An were significantly positively correlated with C_ab_* and a*, and EC, Mv-glu-C, Mv-acglu-vpol, Mv-acglu-vgol, Mv-glu-GC, and GC were significantly positively correlated with L_ab_* and H_ab_*, indicating that these substances have an important influence on wine color (Figure 5A). In order to trace the relationship between these key substances in wine and the flavonoid substances in berries, using these substances (Pt-aclgu, Pt-glu, My-gala, Qu-glu, Qu-rh, T-An, EC, Mv-glu-C, Mv-acglu-vpol, Mv-acglu-vgol, Mv-glu-GC, GC) as the dependent variable, we performed PLSR analysis with the flavonoid compounds in grapes. Seven flavonoid compounds (Pt-acglu, Dp-coglu, T-An, Qu-glu, Pt-glu, Mv-glu-C, EC) in wines were significantly correlated with 27 kinds of substances in grapes (Figure 5C). T-An in grapes was significantly associated with most of the flavonoid compounds in wine except Mv-glu-C. Mv-glu-C was an anthocyanin derivative, mainly connected with C-P-seed and C-P. Interestingly, T-An was also the different compound among the three graft combinations in grapes and wines (Figures 2E, 4E). This showed that the rootstock first influences the accumulation of anthocyanin in the berries, and then the difference in anthocyanin was further reflected in wines, and the difference in anthocyanin concentrations in wine causes the difference in the color parameters (L*, a*, C_ab_*) of the wine. Furthermore, C-P-seed and ECG-P-seed in grapes were also significantly associated with most of the flavonoid compounds in wine, which suggested that they can influence the color of the wine by affecting the key flavonoid compound concentrations in wine.

In addition, PLSR analysis was also used to identify the key environmental factors affecting flavonoid compounds in berries and found that some anthocyanins and flavonols were significantly correlated with sunshine hours (SH; Figure 5B). Flavonols have been shown to be sensitive to light, as indicated by the results of this experiment. This further illustrated the importance of SH on the accumulation of flavonoid substances in the berries. Interestingly, CI and T-rain also affected the concentration of flavonols (My-gala, Ka-gala, and Laricitrins), although not in the same substances as the flavonols affected by SH, suggesting that flavonols were a class of substances that were highly susceptible to regulation by external environmental factors. Temperature only significantly affected the concentrations of Qu-glu, EC-T, and C-P, and HI was significantly associated with isorhamnetins concentrations. This indicated that temperature has a relatively small effect on the flavonoids in grapes compared to light.

In this experiment, we found that 1103P had a higher pruning weight and leaf area, which meant higher vegetative growth, and this vegetative growth suppressed reproductive growth to a certain extent, making ripening slower. The slower ripening rate superimposed on the excessive rainfall for véraison further decreased the accumulation of anthocyanin and flavonol compounds in berries, and this difference was eventually reflected in the resulting wines. For example, the wine of the CS/1103P combination contained fewer anthocyanins and had a higher L* value and lower a*. In contrast, 5A tended to promote ripening, allowing the grapevine to avoid some of the rainfall during the véraison period earlier in rainy vintages, speeding up the accumulation of anthocyanins, this difference was eventually reflected in the wine. Interestingly, it was found that a good vintage (such as 2019) would reduce the differences caused by the rootstock. This was because less rain and more light may provide a favorable growth environment for each of the rootstock combinations, enabling Cabernet Sauvignon to acquire the full-color profile for which it is known. Environmental factors act as signals to affect the expression of certain genes regulating fruit flavonoids, such as *VviFLS* (Sun et al., 2019). It was clear that the final color characteristics of wine were determined by both the rootstock and the vintage, and not by a single factor. The features of a rootstock were enhanced in a specific vintage, and similarly, in other vintages, the differences caused by the rootstock were not apparent, therefore, it was necessary to choose the appropriate rootstock based on the climatic circumstances.

## Conclusion

In China, the use of commercial rootstocks has a relatively short history, and research on the subject has been limited, especially on how rootstocks affect winemaking quality indicators. In addition, eastern viticultural regions have seasonal climates with rainy and cloudy days, where vines are exposed to more stressful conditions than the vines in the Mediterranean climate zone. This provided the motivation to conduct this study, which helps to answer the question of whether rootstocks exhibit unique characteristics in the face of climatic stresses. This experiment showed that 1103P tended to delay the ripening of Cabernet Sauvignon grapes, with lower anthocyanin and flavonol concentrations, and 5A promoted the ripening of Cabernet Sauvignon berries, reduced vine vigor, facilitated the accumulation of anthocyanins in the berries, and produced wines with a high red hue compared with other graft combinations. There was a combined influence between vintages and rootstocks, and rootstock played a key role in flavonoid accumulation in vintages with more rainy and cloudy days. In addition, vintage with sufficient light masked the differences caused by rootstocks. PLSR analysis revealed that sunshine hours during the growing season were a key factor affecting flavonoid compounds and that total anthocyanins were important in affecting wine color, with a significant correlation between grape and wine. In conclusion, considering the influence of rootstocks on flavonoid accumulation in grapes, 5A grafted to Cabernet Sauvignon is recommended in regions with insufficient light and rainy conditions rather than 1103P. In this study, three rootstocks were used to graft Cabernet Sauvignon, and more graft combinations will be investigated in the future to provide various options for the use of rootstocks.

## Data availability statement

The original contributions presented in the study are included in the article/Supplementary material, further inquiries can be directed to the corresponding author.

## Author contributions

XH: formal analysis, data curation, investigation, writing—original draft, and visualization. YW: software and investigation. H-CL, H-QL, X-TG, and X-XP: investigation. FH and C-QD: supervision. JW: conceptualization, writing—review and editing, supervision, project administration, and funding acquisition. All authors contributed to the article and approved the submitted version.

## Funding

This work was supported by the China Agriculture Research System of MOF and MARA (CARS-29).

## Conflict of interest

The authors declare that the research was conducted in the absence of any commercial or financial relationships that could be construed as a potential conflict of interest.

## Publisher’s note

All claims expressed in this article are solely those of the authors and do not necessarily represent those of their affiliated organizations, or those of the publisher, the editors and the reviewers. Any product that may be evaluated in this article, or claim that may be made by its manufacturer, is not guaranteed or endorsed by the publisher.

## Footnotes

^1^ http://cdc.cma.gov.cn/
